# Supplementary material for: Immobilization of Polyethyleneimine (PEI) on Flat Surfaces and Nanoparticles Affects Its Ability to Disrupt Bacterial Membranes
Source: Microorganisms. 2021 Oct 19;9(10):2176. doi: 10.3390/microorganisms9102176 (PMC8540495; doi:10.3390/microorganisms9102176)
Supplement: Supplementary file 1 [file microorganisms-09-02176-s001.zip › microorganisms-1403416-supplementary.pdf]

Supporting Information

Immobilization of Polyethyleneimine on Flat Surfaces and Nanoparticles Affects its Ability to Disrupt Bacterial Membrane

Nesha May O. Andoy[1], Meera Patel[1], Ching Lam Jane Lui[1], Ruby May A. Sullan[1,2,*]

[1] Department of Physical and Environmental Sciences, University of Toronto Scarborough, 1065 Military Trail, Toronto, ON, M1C 1A4, Canada; ruby.sullan@utoronto.ca
[2] Department of Chemistry, University of Toronto, 80 St. George St., Toronto, ON, M5S 3H6, Canada
* Correspondence: ruby.sullan@utoronto.ca

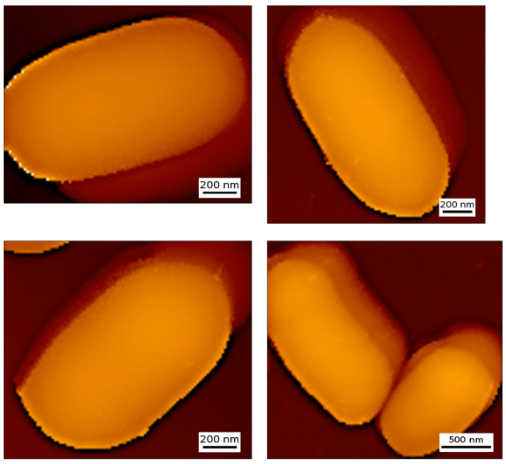

Figure S1. Topography images of *E. coli* immobilized on PEI-coated PC membrane.

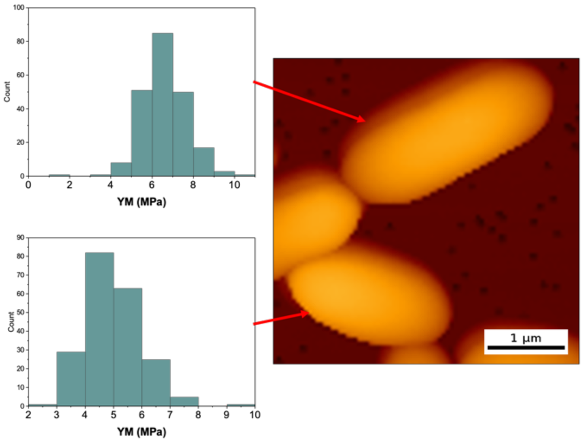

Figure S2. Distribution of Young's modulus obtained for each bacteria using Hertz fit. Only the top portion of the bacteria were selected for obtaining the histogram to avoid potential tip artifacts (also see Figures S10 and S12). Each histogram is then fitted with a Gaussian distribution to obtain the average value for each bacterium. The distribution of these average values is plotted in Figure 1B and the box plots in Figures 2C and 4E. Figure 1B and 2C are from the same data set. Figure 4E is taken from a different data set.

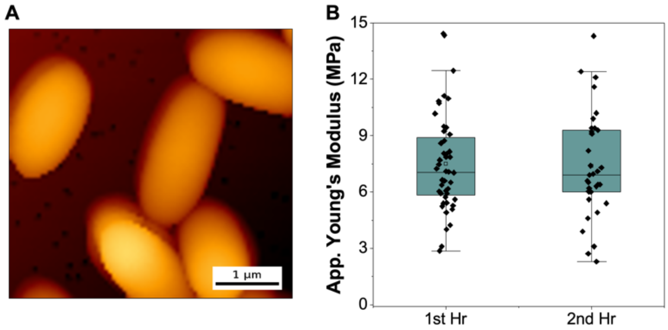

Figure S3. (A) AFM topography images of *E. coli* ~120 mins after immobilization. Similar to Figure S1, no structural changes were observed after 2 hrs of incubation. (B) Young's modulus of *E. coli* immobilized on PC-PEI. Data on the left were obtained within the first 60 min after surface immobilization (1st Hr, n = 48) while data on the right (from a different sample) were obtained during the second 60 min after surface immobilization (2nd Hr, n=33).

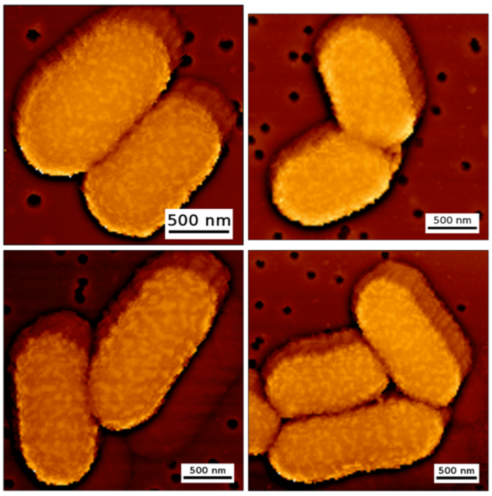

Figure S4. Topography images of *E. coli* pre-treated with 4 µg/ml PEI before surface immobilization. The bacteria were immobilized on PEI-coated PC membrane.

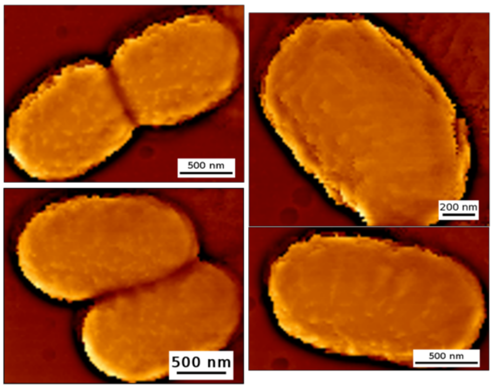

Figure S5. Topography images of *E. coli* pre-treated with 100 µg/ml PEI before surface immobilization. The bacteria were immobilized on bare PC membrane.

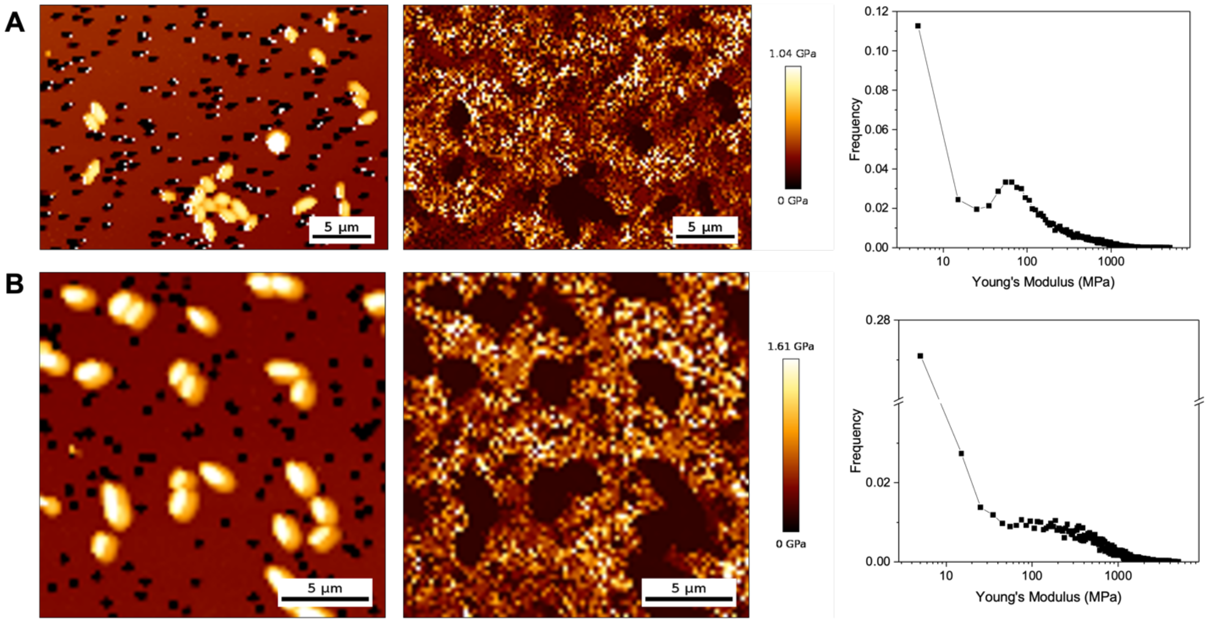

Figure S6. (A) Surface topography (left panel), with its corresponding Young's modulus map (middle panel) and the normalize histogram of the Young's modulus values (right panel) of (A) PC and (B) PC-PEI membrane fixed on top of a glass slide. PEI-treated (100 µg/ml) *E. coli* are shown immobilized on PC-only membrane (A), while *E. coli* (non-treated) are shown immobilized on PEI-coated membrane (B). The apparent elasticity measured on the bare PC surface did not significantly changed upon coating with PEI (rightmost panels), with values ranging from tens to hundreds of MPa. Note that lower elasticity values are those obtained from the surface of the bacteria and holes on the porous membrane.

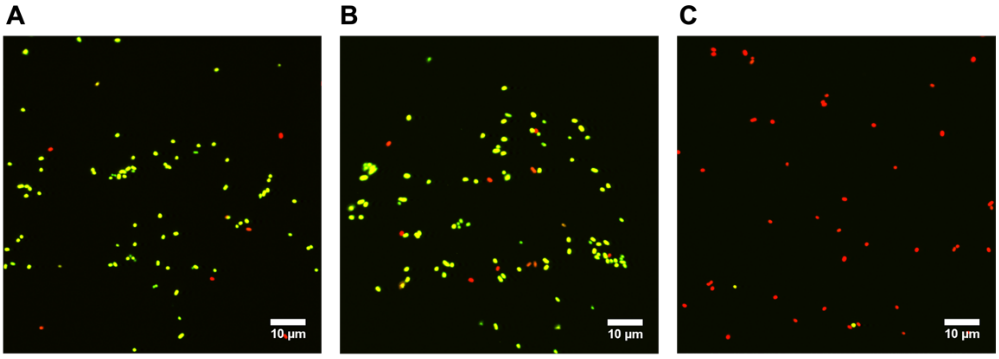

Figure S7. SYTO9/PI staining of *E. coli* (A) before and (B) after 15 min exposure to 4 µg/ml and (C) 100 µg/ml unbound PEI. Note: The concentration of SYTO9 and PI here is 4× higher than in Figure S14 and Figure 1 in the main text.

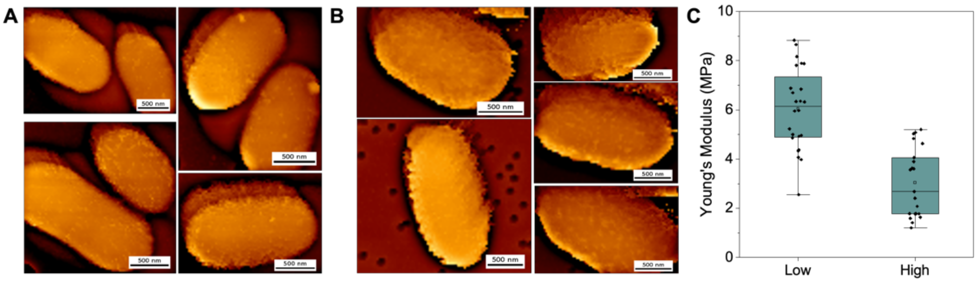

Figure S8. Effect of PEI on the pre-immobilized *E. coli* (AR3110). Surface topography of PEI-immobilized bacteria after 15-minute incubation with (A) 4 µg/ml (Low) and (B) 100 µg/ml (High) PEI concentration, with their corresponding Young's modulus (C).

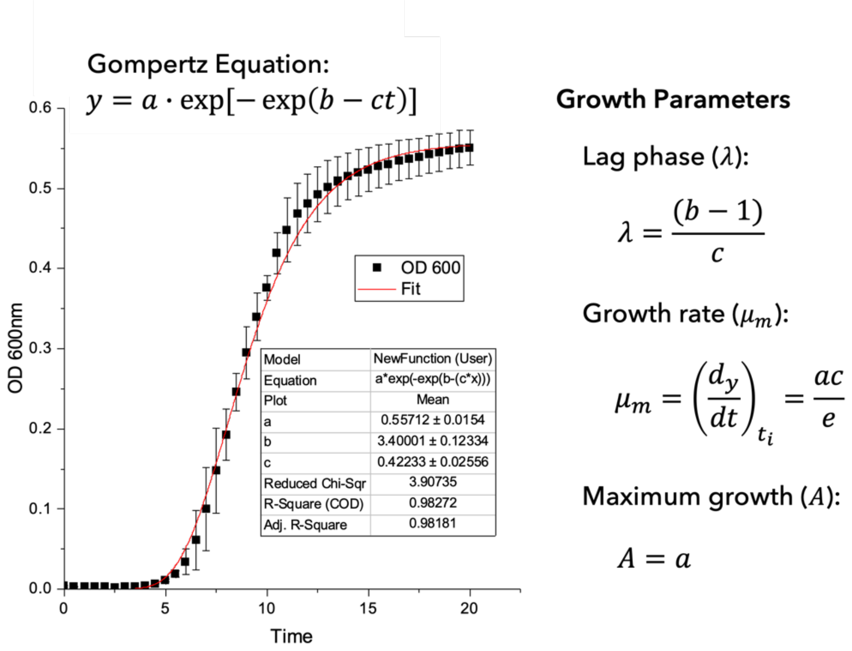

Figure S9. Gompertz fitting of the growth curve and how the growth parameters (lag phase, growth rate, and maximal growth) were calculated from the fit parameters (a, b, and c) of the Gompertz equation, $y = a \cdot \exp\,[-\exp(b - ct)]$.

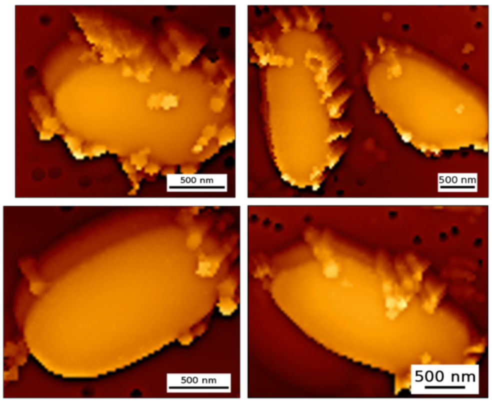

Figure S10. Topography images of *E. coli* pre-treated with 4 µg/ml PDNP-PEI before surface immobilization. The bacteria were immobilized on PEI-coated PC membrane.

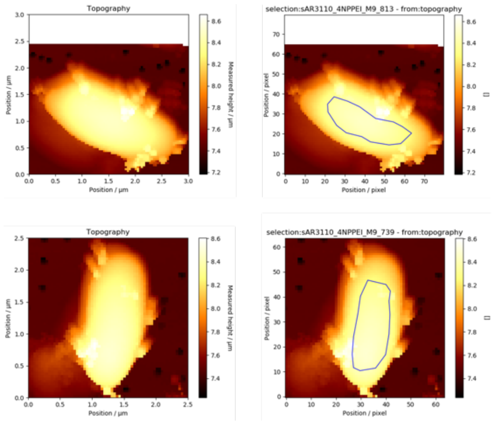

Figure S11. Areas within the bacteria chosen for Young's Modulus determination. Since the nanoparticles are only sparsely distributed, we were able to choose only the areas without nanoparticles to be included in the determination of YM for each bacterium.

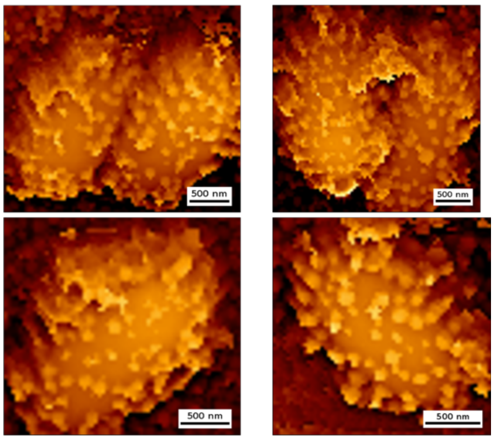

Figure S12. Topography images of *E. coli* pre-treated with 100 µg/ml PDNP-PEI before surface immobilization. The bacteria were immobilized on bare PC membrane.

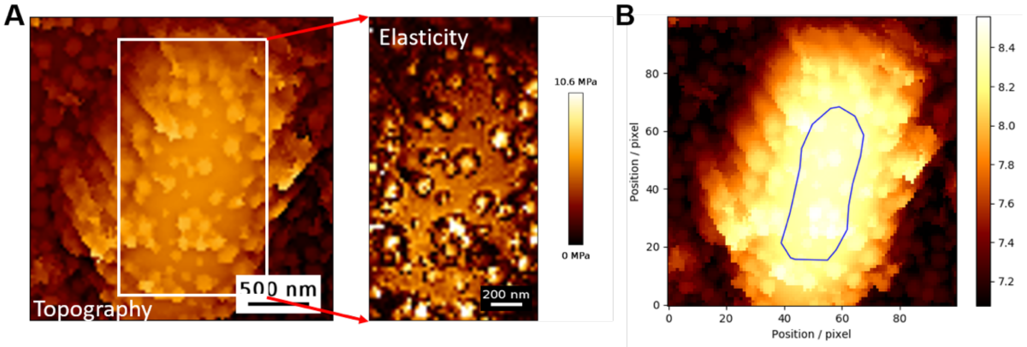

Figure S13. (A) Topography image of *E. coli* with its corresponding elasticity map. (B) Area selected for Young's modulus analysis.

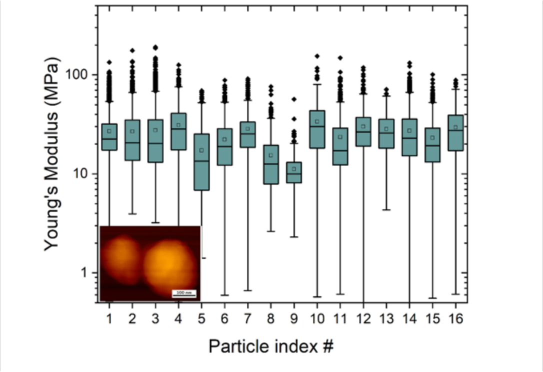

Figure S14. Young's modulus and surface topography (inset) of PEI-coated nanoparticles (PDNP-PEI).

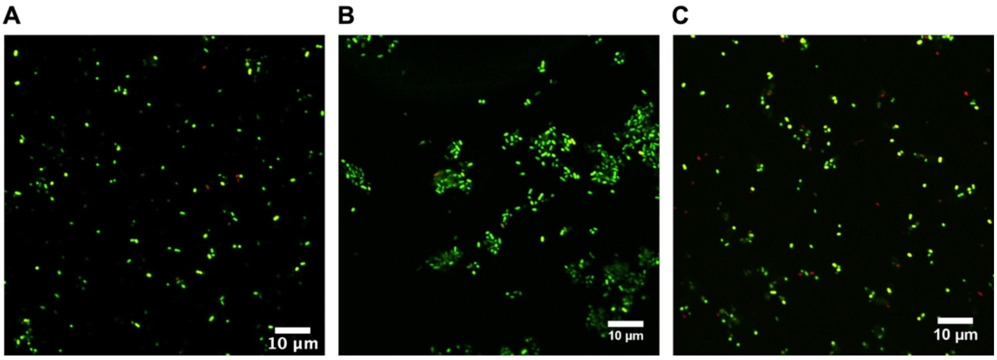

Figure S15. SYTO9/PI staining of *E. coli* (A) before and (B) after 15 min exposure to 4 μg/ml (B) and 100 μg/ml PDNP-PEI.

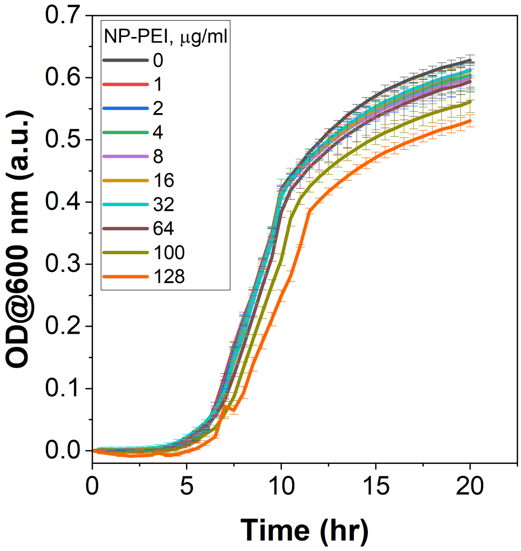

Figure S16. Growth of PDNP-PEI treated *E. coli* in M9 minimal media.
